# Benzydamine—An Affordable Over-the-Counter Drug with Psychoactive Properties—From Chemical Structure to Possible Pharmacological Properties

**DOI:** 10.3390/ph16040566

**Published:** 2023-04-10

**Authors:** Bianca-Eugenia Ősz, George Jîtcă, Andreea Sălcudean, Carmen Maria Rusz, Camil-Eugen Vari

**Affiliations:** 1 Department of Pharmacology and Clinical Pharmacy, Faculty of Pharmacy, George Emil Palade University of Medicine, Pharmacy, Science, and Technology of Targu Mures, 540139 Targu Mures, Romania; bianca.osz@umfst.ro (B.-E.Ő.); camil.vari@umfst.ro (C.-E.V.); 2 Department of Ethics and Social Sciences, Faculty of Medicine, George Emil Palade University of Medicine, Pharmacy, Science, and Technology of Targu Mures, 540139 Targu Mures, Romania; andreea.salcudean@umfst.ro; 3 Doctoral School of Medicine and Pharmacy, I.O.S.U.D, George Emil Palade University of Medicine, Pharmacy, Science and Technology of Targu Mures, 540139 Targu Mures, Romania; carmenrusz20@gmail.com

**Keywords:** benzydamine, neuropathic pain, inflammation, psychotropic, over the counter

## Abstract

Benzydamine is a non-steroidal anti-inflammatory drug with distinct pharmacological properties from other compounds in the same therapeutic class. The differences are structural and pharmacological in nature; the anti-inflammatory mechanism is not strictly explained by the ability to interfere with the synthesis of prostaglandins. The compound is used strictly in local inflammatory diseases (inflammation in the oral and vaginal mucosa). In addition to the therapeutic indications found in the summary of product characteristics (SPC), the compound is used, in high doses, as a psychotropic substance for oral administration, having similar properties to lysergic acid diethylamide (LSD). As an over-the-counter (OTC) compound, it is easy to obtain, and the consequences of using it for purposes other than those assumed by the manufacturer raise various concerns. The reasons are related to the pharmacodynamic and pharmaco-toxicological properties, since neither the mechanism of action nor the possible side effects that would result from systemic consumption, in high doses, even occasionally, have been fully elucidated. The present review aims to analyze the pharmacodynamic properties of benzydamine, starting from the chemical structure, by comparison with structurally similar compounds registered in therapy (as an anti-inflammatory or analgesic) or used for recreational purposes.

## 1. Introduction

Benzydamine, 3-(1-benzyl-1H-indazol-3-yloxy)-N, N-dimethylpropan-1-amine, is a compound with anti-inflammatory action used for the symptomatic treatment of vaginal and oropharyngeal conditions. Pharmaceutical products registered in the European Union containing benzydamine (mouthwashes, oral sprays, vaginal solutions, vaginal solution powder, etc.) are intended only for local use (see Table 1) [1].

In recent years, medicinal products have been diverted and used for recreational purposes. Ingestion of benzydamine hydrochloride-containing products, a route of administration not recommended for therapeutical purposes, has already been reported in young people [2,3,4,5,6]. After oral administration, in doses of 1000–3000 mg, benzydamine produces hallucinations and nonspecific sensory changes, described as trips [4,7]. A case report published in 2019 also suggests that psychotic episodes may persist even after discontinued administration. According to a case report on a young person, the presence of psychotic episodes (insomnia, fear, hallucinations) with immediate onset, after the first consumption of benzydamine in doses of 5000 mg orally, and after occasional consumption for 3 months, were still present even 3 months after the last intake of benzydamine [5].

Since the hallucinogenic effect of benzydamine occurs at doses exceeding 1000 mg, they can be most easily achieved by ingesting pharmaceutical products in the form of vaginal granules or powders.

However, accidental exposure caused by confusion about the correct route of administration must also be considered. The risk of hazardous exposure increases with the number of packages sold. The increase in the number of units dispensed from pharmacies, in the case of over-the-counter (OTC) drugs, may result from television advertising campaigns. In Italy, after the reclassification of preparations containing benzydamine powder from pharmacy-supervised drugs (for which advertising is prohibited) to OTC, a 3-fold increase in the number of packages sold/day was observed during the advertising period compared to pre-advertising. During the advertising period, the number of accidental oral exposures to benzydamine increased due to the misleading TV commercial [8].

Despite the fact that benzydamine is recommended only for topical use, it is rapidly absorbed from the intestine after ingestion. It has a half-life of 7–8 h, undergoes hepatic metabolism, and has renal/biliary excretion. The bioavailability after oral administration is 87%, but absorption after topical application on the skin or mucous membranes is negligible [9].

### 1.1. The Therapeutic Effects of Benzydamine

The anti-inflammatory and analgesic effect of benzydamine have been acknowledged in the literature, with preclinical and clinical studies proving this action even after topical application, as shown in Table 2 and Table 3.

In addition to these studies, there are also numerous systematic reviews in the literature that confirm the effectiveness of benzydamine in the management of oral mucositis after cancer therapy [23,24,25,26,27], sore throat after intubation [28] or pericoronitis [29].

### 1.2. The Side Effects of Benzydamine

Although side effects reported after topical use are well documented, there is a lack of information regarding the toxicity of benzydamine after oral intake. When used topically, on mucous membranes, it is usually well tolerated; reported side effects are numbness, burning, and stinging [30]. After unintentional oral exposure, vertigo, oropharyngeal irritation, pharyngeal pain, vomiting, nausea, pyrosis, hallucinations, and headache have been reported [8]. Central excitatory side effects, like insomnia, fear, and hallucinations, occur at doses exceeding 500 mg/dose [5].

All data on benzydamine side effects come from case reports; there are no official reports on the number of medicinal products sold annually, nor on the number of cases of intoxications (misuse or abuse).

Despite the fact it has been marketed since 1966, the exact mechanisms underlying the anti-inflammatory properties of benzydamine are not fully elucidated, much less the mechanisms underlying its hallucinogenic potential. This review aims to explain, on a theoretical basis, the pharmacological properties of benzydamine by comparison with structurally related compounds registered in therapy. At the same time, the possible mechanisms that could explain the hallucinogenic potential of benzydamine will be proposed, based on the pharmacodynamic properties of some structurally related compounds used for recreational purposes.

## 2. Benzydamine-Proven and Potential Pharmacological Effects

### 2.1. Benzydamine as a Non-Steroidal Anti-Inflammatory Drug

**Figure 1:**
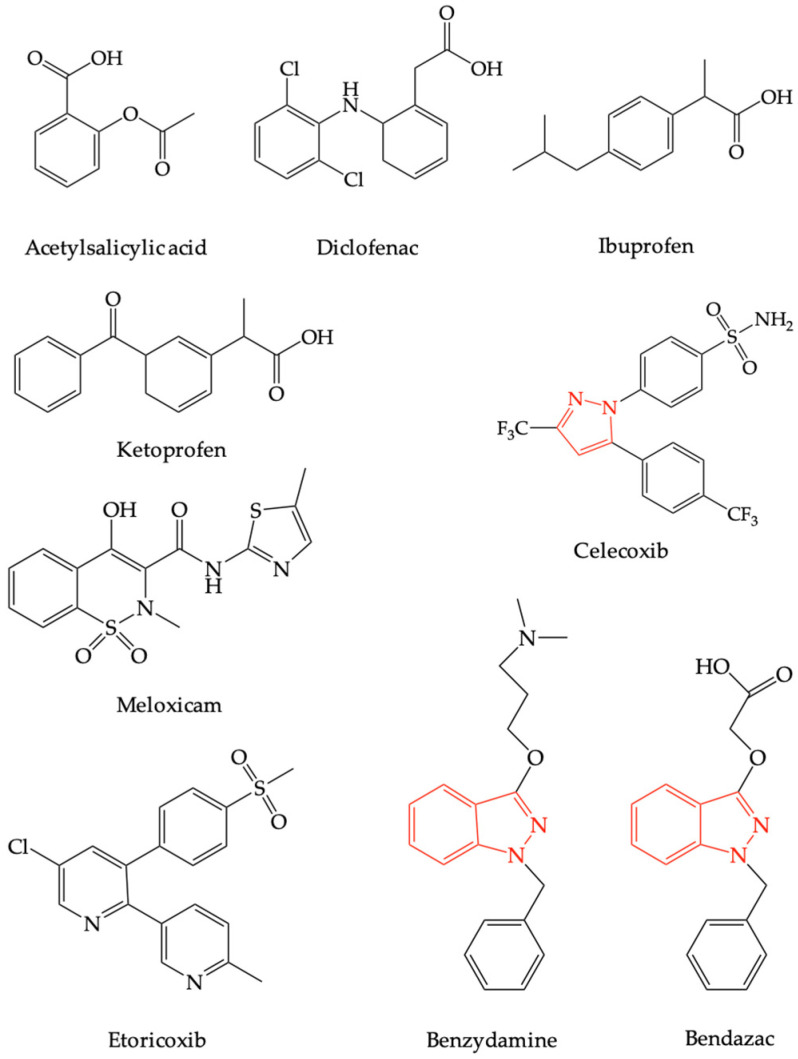
The chemical structures of the most frequently used non-steroidal anti-inflammatory drugs, bendazac and benzydamine.

Benzydamine is a non-steroidal anti-inflammatory drug (NSAID) with completely different structure than other NSAIDs recorded in therapy, with celecoxib as the only relatively structurally related compound because it possesses a pyrazole nucleus [31]. Thus, the only structurally related compound used in therapy for the same therapeutic indications (anti-inflammatory) and which is strictly local, in the form of eye drops, is bendazac (see Figure 1) [32].

It is precisely these structural differences that could explain the different mechanism of action of benzydamine from the other compounds. If, in the case of NSAIDs, the anti-inflammatory effect occurs as a result of a decrease in prostaglandin E2 synthesis (PGE2) following cyclooxygenase-2 (COX-2) inhibition, this mechanism has not been described as occurring in the case of benzydamine [33].

**Figure 2:**
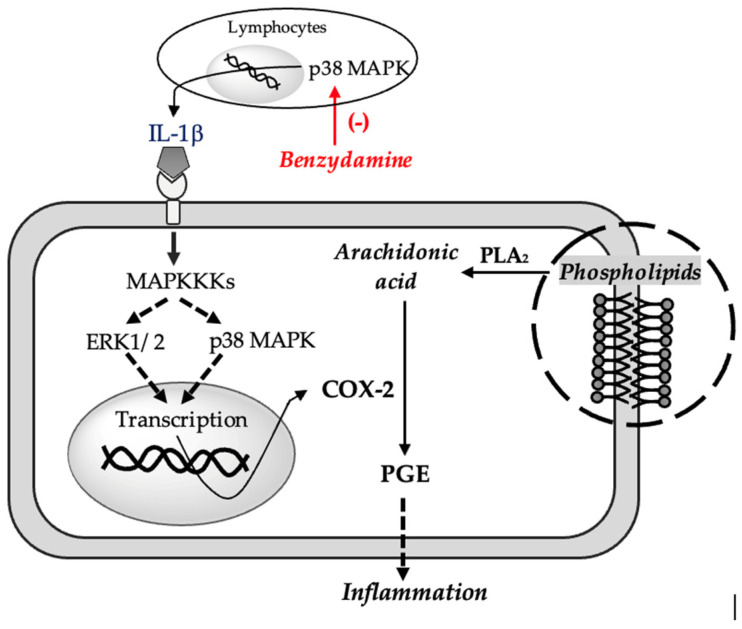
Proposed mechanism for the anti-inflamatory effect of benzydamine. Tumor Necrosis Factor α (TNFα), Interleukin 1β (IL-1β), Mitogen-activated protein kinase (p38 MAPK), Extracellular signal-regulated kinase 1/2 (ERK1/2), Mitogen-activated protein kinase kinase kinase (MAPKKK), Phospholipase A_2_ (PLA2), Prostaglandin E (PGE), Cyclooxygenase 2 (COX-2).

In-vitro studies using human and mouse mononuclear cells have shown that benzydamine prevents the release of pro-inflammatory cytokines (especially TNFα and IL-1β) without significantly influencing the synthesis of inflammatory cytokines (IL-6, IL-8) [34], anti-inflammatory (IL-10) and interleukin-1 receptor antagonist (IL-1RA) following exposure to activator of monocytes and macrophages (e.g., lipopolysaccharide) [33]. This decrease in the synthesis of pro-inflammatory cytokines, however, leads to a decrease in COX-2 activity, taking into account that the enzyme is activated by IL-1 extracellular signal-regulated kinase (ERK1/2) and mitogen-activated protein kinase (p38 MAPK) cascades [35]. A decrease in COX-2 activity should contribute to a decrease in the synthesis of PGE2 mediators that in turn increase COX-2 expression by phosphorylation of p38 [36] (see Figure 2). More recent studies have shown that benzydamine can inhibit monocyte migration and block the p38 MAPK pathway, a key regulator of pro-inflammatory cytokines biosynthesis [37].

The advantage of benzydamine over other NSAIDs is its basic character (the rest of the anti-inflammatories having an acidic character), which allows it to accumulate in inflamed tissues where the pH is more acidic [38].

In conclusion, although limited, the evidence in the literature supports the theory that the anti-inflammatory action of benzydamine is due to the inhibition of the synthesis of pro-inflammatory cytokines by the p38 MAPK pathway and the inhibition of chemotactic cell migration. A decrease in the level of pro-inflammatory cytokines prevents the activation of COX-2, the anti-inflammatory effect being ultimately related to a decrease in COX-2 activity, with consequences similar to the direct inhibition of this enzyme pathway, more precisely the synthesis of PGE2.

### 2.2. Benzydamine as Serotonin Receptor Modulator

**Figure 3:**
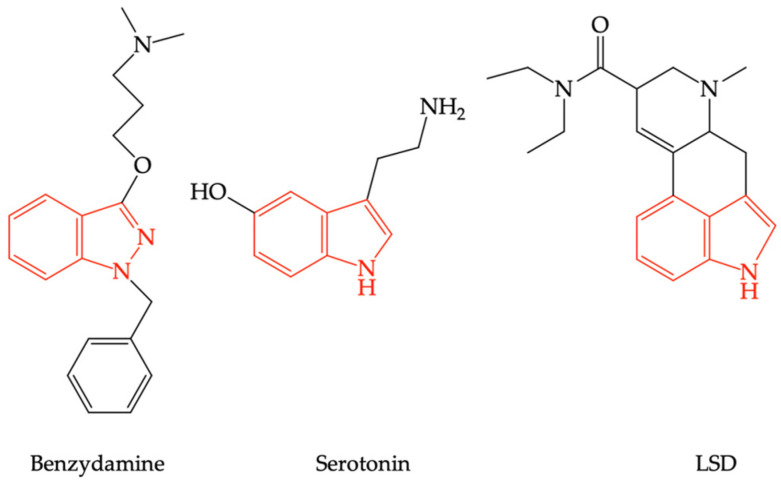
The structural similarities between serotonin, lysergic acid diethylamide (LSD), and benzydamine.

Due to the structural analogy between benzydamine and lysergic acid diethylamide (LSD) (see Figure 3), it has been speculated that benzydamine could act as a serotoninergic 5-HT_2A_ receptor agonist (5-HT_2A_R) [39].

Nowadays, based on the conclusions of some studies that determined how 5-HT_2A_R agonists and antagonists alter the release of dopamine in the mesolimbic system, the theory that 5-HT_2A_R activation enhances dopaminergic neurotransmission in the mesolimbic system is accepted [40]. The hallucinogenic effect of 5-HT_2A_ agonists is supported by this theory [41]. The involvement of 5-HT_2_R in the mechanism of action of psychotropic substances has been speculated since the beginning of the 1980s, with a significant correlation between the binding affinity of psychotropic substances to these receptors and their hallucinogenic properties being observed [42]. The theory was confirmed, however, only after obtaining some 5-HT_2A_R knockout mice in the early 2000s, when it was shown that the hallucinogenic response of substances is different depending on the presence or absence of this receptor subtype [43].

**Figure 4:**
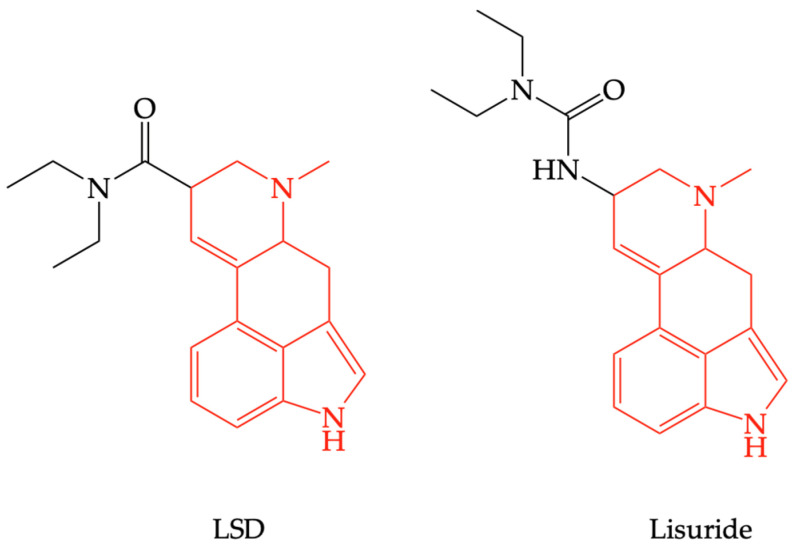
The structural similarity between lysergic acid diethylamide (LSD) and lisuride.

There are some question marks over the consequent effects of 5-HT2AR stimulation, since, in therapy, there are registered 5-HT2AR agonists with no psychotropic effects, such as lisuride [44], which have compounds structurally related to LSD (see Figure 4). This is possible because of differences in intracellular signal transduction consecutive to receptor stimulation, a phenomenon known as “biased agonism”. Thus, being Gq/11-protein-coupled receptor, 5-HT2AR stimulation increases phospholipase C (PLC) activity, an enzyme that hydrolyzes phosphatidyl inositol diphosphate (PIP2) into inositol triphosphate (IP3) and diacylglycerol (DAG); IP3 mobilizes calcium from the endoplasmic reticulum, increasing cytosolic calcium concentration, which could explain the central excitatory effects [45]. Judgment disorders that occur in schizophrenia or after the use of psychotropic substances surface as a result of changes in sensorimotor integration and decision-making [46].

In the regions of sensorimotor integration, 5-HT_2A_R interacts with dopamine D_2_ receptors (D_2_R), with stimulation of serotonin receptors contributing to an increase in dopamine concentration in the prefrontal cortex [47] and in the ventral striatum, more precisely the nucleus accumbens [48]. The increase in dopamine levels in the ventral striatum, a component of the reward system, is associated with the addictive behavior of psychotropic substances [49]. However, at least in the case of LSD, no adverse effects of a psychic nature, nor withdrawal syndrome, were reported upon discontinuation of administration [50]. It is difficult to assess, in the absence of evidence, whether the use of benzydamine produces psychological dependence, or if it behaves similarly to LSD. What has been reported in the literature, however, is the ability of benzydamine to produce psychotic effects even after discontinuation of administration [5]. In addition to this main mechanism, additional mechanisms have been identified that involve the synthesis of arachidonic acid following the activation of phospholipase A2 (PLA2) after 5-HT_2A_R stimulation. In this case, the transduction of the intracellular signal is controlled by means of G_i/o_ proteins and activation of Ras-Raf-MEK-ERK signaling cascade [51]. Because increased PLA_2_ activity has been identified in drug-free patients with schizophrenia [52], this 5-HT_2A_R-mediated transduction pathway, coupled with G_i/o_, is possibly involved in the appearance of hallucinations. The role of the PLA_2_ is to degrade fatty acids from membrane phospholipids that enter myelin (70% of the dry weight is represented by lipids) [53]. Increased activity of PLA2 contributes to an increase in the synthesis of arachidonic acid, from which prostaglandins are formed in the COX-2 pathway. Prostaglandins of series 2 (PGE2) are mediators of inflammation and contribute to an increase in IL-6 expression in patients with schizophrenia (increase also seen in human studies). Since IL-6 can exert neurotoxic effects, an increased level of this cytokine can inhibit neurogenesis [54].

5-HT_2A_R can also be coupled with G_12/13_, leading to the appearance of long-term structural cellular changes because their stimulation activates the Rho-GEF pathway [45,51]. The Rho family is a small GTPases subfamily of Ras superfamily of guanine nucleotide-binding proteins [55] involved in the control of important processes, such as cell division, cytoskeleton rearrangements, cell motility, and neuronal morphogenesis regulation (axon growth, guidance, and branching) [56,57]. In the inactive state, Rho proteins are GDP-bound, their activation requiring activation to the GTP-bound form. One of the cellular regulators that accelerate the conversion of GDP into GTP are guanine nucleotide exchange factors (GEFs) [58].

In conclusion, as a potential agonist of 5-HT_2A_R, benzydamine could cause an increase in the level of dopamine within the reward system, with the possibility of instating addictive behavior.

### 2.3. Benzydamine as a Cannabinoid (CB1) Receptor Modulator

The endocannabinoid system, whose mediators are 2-arachidonoylglycerol and anandamide, is involved in various physiological processes as a result of action on CB1 (CB1R) and CB2 (CB2R) receptors. The localization of the two receptor subtypes in the body is different, CB1R being found predominantly in the central nervous system (CNS), while the distribution of CB2R is predominant in the periphery and spinal cord, which explains the multitude of effects of endogenous cannabinoids, both central and peripheral effects (analgesic, anti-inflammatory) [59].

Autoradiography studies using a radiolabeled synthetic cannabinoid [^3^H] CP55, 940 showed that the density of central cannabinoid receptors is increased globus pallidus, substantia nigra pars reticulata, cerebellum, hippocampal dentate gyrus, cerebral cortex, and striatum. The presence of cannabinoid receptors in the striatum suggests the involvement of these receptors in dopamine reward circuits [60].

#### 2.3.1. The Psychotropic Properties of Benzydamine Linked to Cannabinoid Receptor Type 1 (CB1R)

Recent studies have shown that, in the presence of an antagonist (AM251) of CB1R receptors, long-term depression-like responses after administration of benzydamine were significantly reduced; these observations suggest that benzydamine possesses a cannabinoid mechanism of action [61].

Endocannabinoids via CB1R are involved in maintaining neuroplasticity and neuronal excitability [62]. CB1R stimulation has been shown to contribute to the psychomimetic effects of substances already used for hallucinogenic purposes, such as delta-9-tetrahydrocannabinol (Δ^9^-THC) [63].

The structural similarity between benzydamine and other synthetic agonistic derivatives of CB1R could be the aminoalkylindole group in the structure of synthetic cannabinoids. A similar structural compound, pravadoline, was synthesized by the Sterling Research Group in the early 1990s as a potential NSAID. In preclinical studies, the compound was shown to have analgesic but not anti-inflammatory properties; subsequent studies demonstrated that it does not interfere with prostaglandin synthesis, like other NSAIDs, and that its antinociceptive action is due to CB1R stimulation. Based on these observations, despite the fact that the studies for pravadoline were discontinued, attempts were made to obtain structurally similar analgesics with CB1R affinity [64].

**Figure 5:**
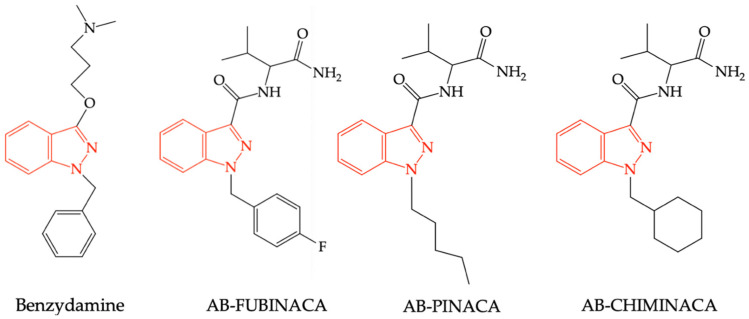
Structural similarities between benzydamine, AB-FUBINACA, AB-PINACA, and AB-CHIMINACA.

Another synthetic cannabinoid, an indazole derivative, similar to benzydamine, was synthesized by the Pfizer company in 2009 as a potential analgesic. AB-FUBINACA has been shown to have an increased affinity for CB1R, acting as an agonist of these receptors, similarly to other structurally related compounds such as AB-PINACA and AB–CHIMINACA, respectively (see Figure 5). Due to adverse reactions of these compounds observed in rodent studies (catalepsy, hypothermia, dose-dependent and inconsistent effect on locomotor activity, agitation, anxiety), as well as those reported in humans (agitation, anxiety, hallucinations), these substances have never been recorded in therapy. However, the previously mentioned compounds are found as “impurities” in various trafficked preparations, especially on the black market, or used as street drugs [65].

CB1R are located in presynaptic neurons in various regions of the CNS, such as the cerebral cortex, hippocampus, hypothalamus, cerebellum, globus pallidus, and mesencephalic periaqueductal gray matter. CB1R receptors are coupled to Gi/o proteins and their stimulation causes the inhibition of adenylate cyclase (AC) activity, which causes a decrease in the level of cyclic adenosine monophosphate (cAMP), the closing of calcium channels, the opening of potassium channels [66], respectively, and the activation of p38 MAPK [67]. Endocannabinoids are synthesized in postsynaptic neurons and released by exocytosis in the synaptic cleft. They act as retrograde inhibitors and control the release of other mediators as a result of the agonist action on CB1R located in presynaptic neurons. [68]. Studies have shown that CB1R agonists cause an increase in dopamine concentration in the striatum. This can be explained by the presence of CB1R in GABAergic interneurons. These interneurons, through gamma-amino-butyric acid (GABA) mediator with inhibitory role, control the release of dopamine in different areas of the CNS. Once CB1R is stimulated, GABA release is prevented, producing a disinhibition on dopaminergic neurons with increased neurotransmission and the appearance of hallucinations [69,70].

The role of p38 MAPK pathway in the pathogenesis of depression, anxiety and addictive behavior has been demonstrated in preclinical studies, but also in the human species [71], because, in this way, pro-inflammatory and inflammatory cytokines are synthesized and have negative consequences on neuronal integrity and functions [72].

Based on these observations, it is difficult to judge how the psychotropic potential of benzydamine can be associated with this mechanism, because it has been described as an inhibitor of p38 MAPK [37]. Even if this property has been observed outside the CNS, the effect cannot be neglected, but further studies are needed to confirm this property at the neuronal level. In addition, CB2R agonists (located in microglia) decrease the activity of p38 MAPK and pro-inflammatory cytokines in a mouse model of Alzheimer’s disease [73].

#### 2.3.2. Analgesic Properties of Benzydamine Linked to Cannabinoid Receptor Type 2 (CB2R)

The ability of medicinal cannabis and cannabis-based medicines to relieve neuropathic pain has been evaluated and confirmed in the human species [74].

Of the synthetic cannabinoids, indazole derivatives appear to have an increased affinity, including for CB2R [75]. The antinociceptive action could be explained by the release of endogenous opioids (β-endorphins) in the periphery, which act agonistically on μ-opioid receptors in primary afferent neurons, thus preventing nociception [76]. However, this mechanism does not fully explain the efficacy of synthetic cannabinoids in relieving neuropathic pain since, in this type of pain, high doses of opioid analgesics are required as an effective treatment [77], which explains the lack of central effects [76].

### 2.4. Benzydamine as Local Anesthetic

Local anesthetics are substances capable of temporarily and reversibly combating painful sensitivity as a result of blockage of voltage-dependent sodium channels in peripheral nerves. There are nine types of voltage-gated sodium channels (Navs), numbered Nav1.1 to Nav1.9; of these, in the peripheral nervous system, the predominant types are Nav1.7, Nav1.8 and Nav1.9 [78]. In addition, the presence of Nav1.1–1.3 and Nav1.6–1.9 has been identified in the human dorsal root ganglion, neurons associated with neuropathic pain, noting that the upregulation of Nav1.7 contributes to the maintenance of this type of pain [79]. Precisely for these reasons, selective inhibitors Nav1.7 (3-Aryl-indole and 3-aryl-indazole derivatives) have been synthesized as analgesics aimed at combating neuropathic pain. Both types of compounds have been shown to be potent inhibitors of Nav1.7 [80], and the question arises whether benzydamine, as an indazole derivative, would possess the same pharmacodynamic profile, even if it is completely structurally different from classical local anesthetics, which are derivatives of esters or amides. Moreover, the application of benzydamine as a gel or spray on endotracheal tube cuff has been shown to significantly reduce the incidence of postoperative sore throat, hoarseness of voice or cough, the effect being superior to other local anesthetics (e.g., lidocaine) [81,82,83].

In addition to inhibition of Navs, in the case of benzydamine, inhibition of nervous excitability by direct or indirect action on acid-sensing ion channels (ASICs) or transient receptor potential (TRP) cannot be excluded [84].

ASICs represents an H^+^-gated subgroup of the degenerin/epithelial sodium channel (DEG/ENaC) family of proteins, ion channels expressed at the level of peripheral sensory neurons, as well as at the level of spinal nociceptive pathways. These channels are sensitive to physiological or pathological variations in pH and are activated by the extracellular proton. Tissue damage and inflammation cause a decrease in local pH with activation of ASICs, increased permeability for Na^+^ and/or Ca^2+^, and stimulation of nerve impulse transmission along nociceptive pathways [85,86]. Since there are studies that have demonstrated the importance of these channels in nociception, noting that their inhibition contributes to a decrease in pain intensity in various pain syndromes, this mechanism of action should not be neglected [87,88,89,90].

TRP channels are expressed in sensory nerves that innervate peripheral tissues and are activated by chemical agonists, mechanical stimulation, or temperature changes. Activation involves opening Ca^2+^ channels with consequent release of neuropeptides (CGRP-calcitonin gene-related peptides, substance P or NKA—neurokinin A) [91], molecules involved in the transmission and perception of pain, regardless of their nature [92]. Therefore, to explain the analgesic effect of benzydamine, the opening of TRP channels should be prevented.

## 3. Conclusions

In conclusion, starting strictly from the chemical structure of benzydamine and the pharmacological properties reported in the literature, both for the substance itself and for structurally related compounds, its analgesic, anti-inflammatory, local anesthetic, and psychotropic effects can be explained as follows:

- The analgesic and/or anti-inflammatory effect could be the consequence of agonist action at the level of CB2R, decreased synthesis of pro-inflammatory cytokines (TNFα, IL-1) as a result of inhibition of p38 MAPK pathway, blockade of Nav1.7 of dorsal root ganglia, modulation of the opening of acid-sensing ion channels (ASICs), and/or decreased release of neuropeptides in the periphery as a result of the inflow of the opening state of TRP channels;
- The local anesthetic effect would be due to blocking voltage-gated sodium channels (Navs) in peripheral nerves;
- The psychotropic and hallucinogenic effect occurs as a result of increased dopamine release in the nucleus accumbens, or as a result of 5-HT_2A_R stimulation due to the action on CB_1_R receptors following the suppression of GABA-inhibitory control over the release of dopamine.

These observations must be confirmed by targeted studies on the properties of benzydamine.

Benzydamine is easy to obtain and is often sold without a prescription. To reduce the number of unintentional poisoning or intentional drug misuse cases, a change should be made to the legal status of medicinal products containing benzydamine. Another solution would be to obtain other compounds with similar anti-inflammatory properties with lower lipophilicity and no effects on the central nervous system.

## Figures and Tables

**Table 1:** Authorized medicinal products containing benzydamine in Europe [1].

Medicinal Product	Concentration/Dose	Route of Administration	Member State Where Product Is Authorized *
Solution (spray)	0.30%	Oral mucosa	BG, HU, PL, GR, FI, SE, NO, SK, HR, CZ, RO, BE, DE, AT, XI
Solution (spray)	0.15%	Oral mucosa	BG, IT, DE, SK, RO, ES, UK, IE, SI, CZ, SK, PL, SE, NO, DK, LT, HR, MT, IS, HU, BE, AT, XI
Solution (mouthwash)	0.30%	Oral mucosa	IT, PL
Solution (mouthwash)	0.15%	Oral mucosa	IT, PL, SK, SI, RO, HR, CZ, IE, MT, XI
Solution (vaginal irrigation)	1%	Vaginal mucosa	DE, IT
Solution (vaginal irrigation)	0.1%	Vaginal mucosa	IT, FR
Solution (vaginal irrigation)	140 mg	Vaginal mucosa	SK, CZ, ES
Cream	0.5%	Vaginal mucosa	IT
Tablets	3 mg	Oral mucosa	BG, IT, PL, RO, CZ, LT, HU, SI, GR, LT, AT, SK, AT, DE
Gel	5%	Skin	BG, IT, RO, ES
Cream	5%	Skin	BG, IT, PL
Cream	3%	Skin	NL, IE
Ointment	3%	Skin	ES
Granules for vaginal solution	500 mg	Vaginal mucosa	SK, IT, ES, LT, RO
Powder for vaginal solution	53.2 mg/g	Vaginal mucosa	PL
Toothpaste	0.5%	-	IT

* Austria (AT), Belgium (BE), Bulgaria (BG), Croatia (HR), Czech Republic (CZ), Denmark (DK), Finland (FI), Germany (DE), Greece (GR), Hungary (HU), Iceland (IS), Ireland (IE), Italy (IT), Lithuania (LT), Malta (MT), Netherlands (NL), Northern Ireland (XI), Norway (NO), Poland (PL), Romania (RO), Slovenia (SI), Slovakia (SK), Spain (ES), Sweden (SE), United Kingdom (UK).

**Table 2:** Preclinical evidence of the anti-inflammatory/analgesic potential of benzydamine (studies published in the last 5 years).

Study Design	Animal Species	Dose/Route	Evidence of Efficacy	References
Experimental inflammation (2% formaldehyde)	Rats	100 mg/kg/injection (rat paw)	Inhibition of paw edema	[10]
Experimental oral ulcers (50% acetic acid)	Rats	Not mentioned	Oral ulcers diameter reduction; reduction of TNF-alpha levels	[11]
Experimental buccal mucosal ulcer (99.7% acetic acid)	Hamsters	0.15% benzydamine mouthwash	Oral ulcers healing	[12]

**Table 3:** Clinical evidence of the anti-inflammatory/analgesic potential of benzydamine (studies published in the last 5 years).

Study Design	No. of Participants	Dose/Route	Evidence of Efficacy	References
Multicenter, randomized	60	0.15% benzydamine hydrochloride (mouthwash)	Reducing the severity of oral mucositis (after platinum-based chemotherapy)	[13]
Randomized, placebo-controlled	60	0.045 g, 30 mL oral spray	Reduction of the severity of pericoronitis (after surgery)	[14]
Prospective, two-arm pilot study	32	Not declared	Reduction of throat inflammation (patients with acute tonsillopharyngitis)	[15]
Randomized	36	0.15% benzydamine hydrochloride (mouthwash)	Reduction of postoperative pain after periodontal surgery	[16]
Randomized	20	10 g mouth gel, each g contains: benzydamine hydrochloride 10 mg, cetylpyridinium chloride 1 mg	Reduction in the severity of symptoms in patients with oral lichen planus	[17]
Randomized	60	0.15% benzydamine hydrochloride (mouthwash)	Reduction of oral mucositis severity (after chemoradiotherapy)	[18]
Randomized	80	0.15% benzydamine hydrochloride (mouthwash)	Reduction of oral mucositis severity after neck and head radiotherapy	[19]
Restrospctive	153	0.15% benzydamine hydrochloride (mouthwash gargle)	Alleviating postoperative pain in the neck	[20]
Randomized, double-blind, placebo-controlled	39	0.15% benzydamine hydrochloride (mouthwash gargle)	Reduction in post-tonsillectomy pain and bleeding	[21]
Randomized	129	0.15% benzydamine hydrochloride (mouthwash)	Reduction in the number of inflammatory complications (patients with fractures of the alveolar processes of the jaws)	[22]

## Data Availability

Not applicable.
